# Posterior medial frontal cortex and threat-enhanced religious belief: a replication and extension

**DOI:** 10.1093/scan/nsaa153

**Published:** 2020-11-12

**Authors:** Colin Holbrook, Marco Iacoboni, Chelsea Gordon, Shannon Proksch, Ramesh Balasubramaniam

**Affiliations:** Department of Cognitive and Information Sciences, University of California, Merced, CA 95343, USA; Department of Psychiatry and Biobehavioral Sciences, Semel Institute for Neuroscience and Human Behavior, David Geffen School of Medicine, University of California, Los Angeles, CA 90024, USA; Department of Cognitive and Information Sciences, University of California, Merced, CA 95343, USA; Department of Cognitive and Information Sciences, University of California, Merced, CA 95343, USA; Department of Cognitive and Information Sciences, University of California, Merced, CA 95343, USA

**Keywords:** posterior medial frontal cortex, religiosity, group bias, transcranial magnetic stimulation

## Abstract

Research indicates that the posterior medial frontal cortex (pMFC) functions as a ‘neural alarm’ complex broadly involved in registering threats and helping to muster relevant responses. Holbrook and colleagues investigated whether pMFC similarly mediates ideological threat responses, finding that downregulating pMFC via transcranial magnetic stimulation (TMS) caused (i) less avowed religious belief despite being reminded of death and (ii) less group bias despite encountering a sharp critique of the national in-group. While suggestive, these findings were limited by the absence of a non-threat comparison condition and reliance on sham rather than control TMS. Here, in a pre-registered replication and extension, we downregulated pMFC or a control region (MT/V5) and then primed participants with either a reminder of death or a threat-neutral topic. As mentioned previously, participants reminded of death reported less religious belief when pMFC was downregulated. No such effect of pMFC downregulation was observed in the neutral condition, consistent with construing pMFC as monitoring for salient threats (e.g. death) and helping to recruit ideological responses (e.g. enhanced religious belief). However, no effect of downregulating pMFC on group bias was observed, possibly due to reliance on a collegiate in-group framing rather than a national framing as in the prior study.

As both lived experience and neuroscientific research attest, much of our mental life is characterized by mind-wandering and automatic behavior (Vatansever *et al.*, 2017). Periodically, however, when expectations are violated, goal conflicts are detected or performance errors are committed, we orient and engage in adaptive behavioral adjustments to the eliciting stimulus. Relatedly, models of reinforcement learning typically include the concept of a ‘prediction error’, or an observed discrepancy between an expected outcome and an actual outcome (Schultz, 2006), which signals a need to adjust one’s responses. In the brain, convergent studies indicate that regions of posterior medial frontal cortex (pMFC) function as neural alarms, monitoring for anomalies, discrepancies, errors or concerns of various types and recruiting additional neural resources to make adjustments (Botvinick *et al.*, 2001; Bush *et al.*, 2002; Ridderinkhof, 2004; Ullsperger *et al.*, 2004; Rushworth *et al.*, 2007; Izuma and Adolphs, 2013).

The pMFC appears responsive to a wide array of elicitors (Izuma, 2013). For example, the dorsal anterior cingulate cortex (dACC) component of the pMFC has been implicated in relatively low-level cognitive control functions such as those measured in the Stroop, Flanker, divided attention or go/no-go tasks (Bush *et al.*, 2002). With regard to social cognition, dACC activity has been linked with social exclusion (Eisenberger, 2012) and with the experience of arguing for an incongruent belief (i.e. cognitive dissonance), then updating one’s beliefs, thereby resolving the incongruity (van Veen *et al*., 2009). Similarly, dACC has been hypothesized to evoke intensified commitment to moral or cultural values as a response to threatening stimuli (Proulx *et al.*, 2012; Jonas *et al.*, 2014). The rostral cingulate zone (RCZ; Picard and Strick, 1996), which overlaps with the dACC (Amodio and Frith, 2006), is likewise associated with the production of feedback- and error-related responses, indicating the need for compensatory adjustments (Gehring *et al.*, 1993; Cohen and Ranganath, 2007; Di Pellegrino *et al.*, 2007). With regard to social cognition, RCZ activation tracks tendencies to heighten social conformity following evidence that one’s opinions deviate from group consensus (Klucharev *et al.*, 2009; Izuma and Adolphs, 2013).

Subdivisions of pMFC, including regions beyond dACC/RCZ (e.g. aspects of dorsomedial pre-frontal cortex), perform specialized functions particular to diverse aspects of cognitive control (Amodio and Frith, 2006; Fan *et al.*, 2008; Ullsperger *et al.*, 2014). Nonetheless, as the methods for modulating pMFC utilized in the present experiment do not discriminate between subdivisions, we will refer in a broad sense to pMFC as instantiating an ‘alarm’, which prompts a variety of adjustments to anomalies, discrepancies or threats of various types.

## Downregulating pMFC

Transcranial magnetic stimulation (TMS) stimulates the brain non-invasively by producing a rapidly varying magnetic field over the stimulated subject’s scalp (Fregni and Pascual-Leone, 2007), upregulating or downregulating targeted regions and thereby permitting causal inference about the contribution of that region. In line with construing pMFC as generating an alarm signal that recruits solutions to problems as they arise, experimental downregulation of pMFC activity via TMS reduces choice-induced preference change (Izuma *et al.*, 2015). In this instance, the problem of disagreement between one’s choices and one’s preferences (e.g. having rejected items that were actually liked), which typically leads individuals to update their preferences in line with their choices (e.g. to retrospectively decrease liking of the rejected items), appears causally linked to pMFC: when the capacity to activate pMFC is downregulated, choice-induced preference change diminishes. A similar dynamic has been observed with regard to social conformity. Whereas cues of disagreement with others (the problem) typically inspire shifts in one’s judgment to accord with the majority (the solution), downregulation of pMFC significantly reduces such conformity effects (Klucharev *et al.*, 2011).

A recent study synthesized this work with research indicating that cues of threat can arouse relevant attitudinal responses (Holbrook *et al.*, 2016). In the experiment, all participants were reminded of their own inevitable death and bodily decomposition and then were asked to (i) rate their degree of belief in religious concepts (e.g. Heaven) and (ii) evaluate a recent Latin American immigrant to the USA expressing sharp criticisms of American culture (the sample’s relevant national in-group).^1^ Whereas prior work has primarily focused on relatively low-level alarm-response dynamics, the prospect of death was chosen as an alarm manipulation to assess the contribution of pMFC to the recruitment of relatively high-level ideological responses, building on an extensive literature indicating that death reminders intensify religiosity (e.g. Osarchuk and Tatz, 1973; Norenzayan and Hansen, 2006; Willer, 2009; Tracy *et al.*, 2011; Jong *et al.*, 2012; Vail *et al.*, 2012). The critique of the USA was intended to function as an ideological alarm, to assess the role of pMFC in mustering rejection of out-group perspectives. Indeed, downregulating pMFC significantly decreased both avowed religious belief and derogation of the critical out-group member.

While noteworthy as a proof-of-concept demonstration that ideological responses to threats can be experimentally reduced by pMFC downregulation, the design suffered two key limitations. First, because all participants were reminded of death to motivate religious belief (Jong, Halberstadt and Bluemke, 2013), the possibility exists that downregulating pMFC might also reduce avowed religious faith due to some unanticipated link between pMFC and belief in or cognitive representation of religious concepts, rather than an alarm response. Relatedly, although recent work has failed to replicate findings that death reminders accentuate group prejudice (Klein *et al.*, 2019), the absence of a non-threat condition leaves open the possibility that downregulating pMFC reduced group bias due to the conjunction of the critical out-group stimulus and the death prime (e.g. as a ‘double-shot’ alarm). Second, the prior study relied on a sham TMS control manipulation. Although there was no difference in self-reported state affect between the sham and actual TMS conditions, the possibility remains that the difference in physical sensation between sham and actual TMS, which can be uncomfortable, drove the observed effects, rather than reduction in pMFC reactivity. To address these limitations, the present study replicates and extends the design of Holbrook *et al.*, (2016) by (i) adding a control non-death writing condition and (ii) replacing sham with actual stimulation in a control TMS region [lateral temporo-occipital area (MT/V5)].^2^

### Primary predictions

#### Religious belief.

We predicted that downregulatory TMS of pMFC, relative to control stimulation of MT/V5, would decrease positive religious belief in the death reminder condition, but not in the neutral non-threat condition, inasmuch as a pMFC-mediated alarm generates enhanced religious belief as an ideological response to the problem of death, but pMFC does not otherwise appear implicated in religious cognition *per se.* No difference in religiosity was expected between the non-threat prime conditions.

#### Group bias.

We predicted that, relative to control stimulation of MT/V5, a character sharply critiquing a relevant in-group would be rated more positively following downregulation of pMFC. We did not anticipate an effect of the death reminder on group bias, as it was the critique of the in-group which was hypothesized to trigger an alarm reaction (Holbrook *et al.*, 2016) and in light of the failures of replication of ostensible effects of death cues on group bias (Klein *et al.*, 2019).

## Methods

The study was pre-registered after data collection had commenced and prior to the analysis (see https://osf.io/ycjt8/). The full materials, dataset and analysis syntax are available in the Supplemental Online Materials (SOM).

### Participants

Undergraduates at the University of California, Merced (UCM), were recruited for a study, ostensibly consisting of a series of unrelated measures, in exchange for US$15 and two research credits. Participants were pre-screened by e-mail for history of neurological disorders and other contraindications to TMS (see the SOM) as well as for baseline religiosity. Prospective participants who identified either as a ‘very devout believer in God’ or as an atheist were excluded from participating during pre-screening; only those who identified as a ‘moderate believer in God’ or as ‘not sure/agnostic’ were eligible, to ensure that participants would deliberately consider the questions rather than respond habitually. Six participants who indicated that they would like to stop TMS due to discomfort were compensated and excused without penalty. The final sample consisted of 96 participants (63.5% female, *M*_age_ = 20.0 years, *SD* = 1.41).^3^ About 63.5% of the participants identified as Latinx, 11.5% as South Asian, 9.4% as East Asian, 5.2% as Black, 5.2% as White, 2.1% as Middle Eastern and 3.1% as Other. The sample size was based on the samples used in Klucharev *et al.*, (2011) and Holbrook *et al.*, (2016). The study was approved by the UCM Institutional Review Board, and written informed consent was obtained from all participants.

## Design

In a 2 (TMS: pMFC *vs* MT/V5) by 2 (threat: death *vs* neutral) between-subjects design, participants received downregulation of either pMFC or MT/V5, and completed a brief writing task either about their own death or a neutral control topic, their morning routine (see the SOM). Participants then completed several distracter or unrelated tasks before completing measures of state affect, religiosity and group bias.

### Theta burst stimulation

Theta burst stimulation (TBS) is a form of patterned TMS. TBS protocols have been modeled from repetitive electrical stimulation protocols that induced long-term potentiation or long-term depression in animal studies (Huang *et al.*, 2005). Continuous theta burst stimulation (cTBS) reduces activity for approximately 1 h (see Holbrook *et al.*, 2018). Following the procedure used in the original study, we stimulated the right pMFC in the experimental condition: RCZ, Brodmann areas 24, 32, 6 and 9. In the control condition, we stimulated right MT/V5 (see Supplementary Figure S1).

The cTBS protocol was administered using a Magstim Rapid^2^ at target locations in 50 Hz triplets of pulses delivered at 5 Hz intervals over 40 s, for a total of 600 pulses at 80% of the subject’s active motor threshold (AMT). If a subject’s 80% of AMT was a greater intensity that can safely be administered with our system, then we stimulated at the maximum intensity that was safe (e.g. 45% of maximum stimulator output). Due to a number of pilot participants reporting significant pain during cTBS over pMFC—resulting from sensitivity of the stimulation location and the relative subjective intensity of stimulation from the double-cone coil—any participant with an AMT above 40% of our machine’s maximal stimulus output did not undergo cTBS stimulation of pMFC and was excluded from the experiment.

AMT in the pMFC condition was determined as the intensity at which we observed at least 5 out of 10 motor-evoked potentials (MEPs) of at least 100 µV greater than the background noise, measured from the anterior tibialis (AT) using surface-electrode electromyography (EMG) with single-pulse TMS to the AT motor hotspot. For single-pulse TMS, the double-cone coil (Magstim, 2 × 126 mm, Carmarthenshire, UK) was fit over the head and held with the handle vertical to the AT hotspot, with the coil orientation parallel to the anterior–posterior midline. The AT region of primary motor cortex was chosen for motor thresholding because the tibia representation and the pMFC are located at a similar depth within the medial cortex. The location of pMFC was calculated for each participant according to the size of their head, using the international 10–20 system (Klem *et al.*, 1999), as in the original study and in other TMS studies (e.g. Knoch *et al.*, 2009; Klucharev *et al.*, 2011). Using this system, we measured the head and located electrode placement area F2 as the pMFC stimulation site. For cTBS, the double-cone coil was fit over the head with the handle vertical over the pMFC stimulation site, and the coil orientation parallel to the anterior–posterior midline.

AMT in the control condition (MT/V5) was determined as the intensity at which we observed at least 5 out of 10 MEPs of at least 100 µV greater than the background noise, measured from the first dorsal interosseus (FDI) using surface-electrode EMG with single-pulse TMS to the FDI hotspot. For single-pulse TMS, the figure-of-eight coil (Magstim, D702 double 70 mm coil, Carmarthenshire) was placed tangential to the head at an angle of $\sim$45° from the anterior–posterior midline. The FDI region of primary motor cortex was chosen for motor thresholding because the hand representation and MT/V5 are located at a similar depth within the cortex. MT/V5 was also located using the 10–20 system, and electrode area PO8 was the site for coil placement. For cTBS, the figure-of-eight coil was placed tangential to the head, with coil orientation parallel to the anterior–posterior midline. (The full details of the protocol are provided in the SOM.)

Next, participants performed the experimental tasks at a computer station in a nearby room.

### Measures

#### Motor and visual distracter tasks.

Participants first completed a series of filler motor and visual estimation tasks to ensure that downregulation of the pMFC or MT/V5 had taken effect (Huang *et al.*, 2005) and to defray suspicion about the target hypotheses.

#### *Death* vs *control writing task.*

Next, participants were asked to write about their own death and bodily decomposition, a threat induction selected because of the evident link between the prospect of death and reassuring thoughts of an afterlife, or to write about their morning routine as a neutral control (see the SOM for details).

#### Self-reported affect.

Following the writing task, participants completed the Positive and Negative Affect Schedule (PANAS-X; D. Watson and L.A. Clark, unpublished data; see the SOM for details), with affect terms rated according to a five-point Likert scale (1 = ‘Not at All’; 2 = ‘A little’; 3 = ‘Moderately’; 4 = ‘Quite a bit’; 5 = ‘Extremely’).

Participants were next assigned to complete the religiosity and group prejudice measures (random order).

#### Religiosity.

As in the initial study, religious belief was measured using a version of the Supernatural Belief Scale (Jong *et al.*, 2012) modified to create two scales consisting of positive and negative aspects of Western religious belief, comparable to the positive and negative authors in the measure of group bias (see the following subsection). The items were presented in random order and rated according to an eight-point Likert scale (1 = ‘strongly Ddisagree’; 8 = ‘strongly agree’). The positive scale consisted of the following: (i) ‘There exists an all-powerful, all-knowing, loving God’; (ii) ‘There exist good personal spiritual beings, whom we might call angels’ and (iii) ‘Some people will go to Heaven when they die’ (α = 0.95). The negative scale consisted of the following: (i) ‘There exists an evil personal spiritual being, whom we might call the Devil’; (ii) ‘There exist evil, personal spiritual beings, whom we might call demons’ and (iii) ‘Some people will go to Hell when they die’ (α = 0.96).

#### Group bias.

In the original study, participants read two essays ostensibly written by immigrants from Latin America: one of whom praised American society and one of whom harshly condemned American society. This framing would not be feasible using our UCM undergraduate sample, which is predominantly composed of Latinx students, many of whom are from immigrant families, and where most students of all backgrounds identify as allies of immigrant groups. Therefore, we created new essays intended to mirror the previous essays, this time ostensibly written by two transfer students, one of whom praised the UCM community and one of whom sharply criticized the UCM community (see the SOM for the full text). After reading each essay, participants rated their agreement with six statements using the same scale as in the religious belief ratings (a modified version of the Interpersonal Judgment Scale (Byrne, 1971)): (i) ‘I like the person who wrote this’, (ii) ‘I think this person is intelligent’, (iii) ‘This is the kind of person I would like to work with’, (iv) ‘I think this person is honest’, (v) ‘I agree with this person’s views’ and (vi) ‘From my perspective, I think this person’s opinions of UC Merced are true’ (complimentary author: α = 0.89; critical author: α = 0.90).

Finally, participants completed tasks related to a project intended for separate publication, then demographic questions, including an item probing ‘How important is being a UC Merced student to your sense of who you are?’ (1 = ‘Not important to me at all’; 2 = ‘Moderately important to me’; 3 = ‘Extremely important to me’). Once the survey was complete, participants were thanked, compensated and debriefed.

## Results

### Self-reported affect

Consistent with prior literature indicating that the death writing task does not influence conscious affect and with the intended comparability of the degree of discomfort related to TMS of the pMFC and control MT/V5 regions, a multivariate analysis of variance (ANOVA) revealed no significant effects of TMS condition, threat condition, nor interactions between the two, on any of the state affect subscales comprising the PANAS-X (*p*s 0.05–0.98) (see Supplementary Tables S1 and S2). Pooling conditions, participants reported feeling relatively low levels of fear (*M* = 1.59, *SD* = 0.68) or overall negative affect (*M* = 1.48, *SD* = 0.60), and moderately positive overall affect (*M* = 2.36, *SD* = 0.88).

### Religious belief

#### Positive religious beliefs.

**Fig. 1.**
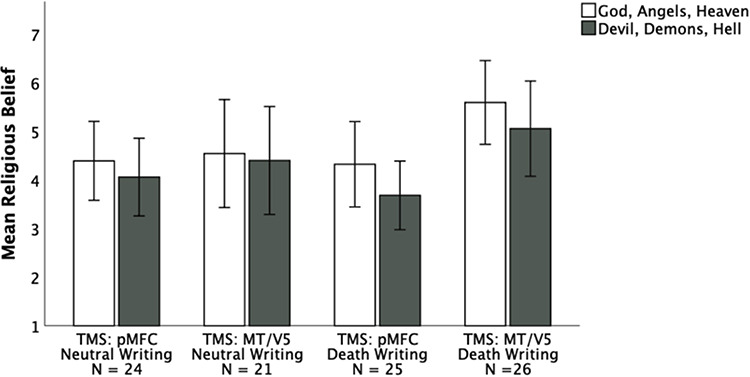
Ratings of positive and negative religious beliefs by TMS downregulation and threat writing condition. Error bars indicate 95% confidence intervals.

As anticipated, planned contrasts between the subsample of participants who wrote about death (i.e. the sample replicating the original study) revealed significantly lower endorsement of positive religious beliefs following pMFC stimulation *vs* MT/V5 stimulation (pMFC death condition: *M* = 4.32, *SD* = 2.13; MT/V5 death condition: *M* = 5.59, *SD* = 2.13), *F*(1, 49) = 4.54, *P* = 0.038, *η_p_^2^ *= 0.05, 95% CI 0.07–2.47 (see Figure 1). Also consistent with an alarm interpretation of pMFC, planned contrasts revealed no effect of TMS in the neutral writing condition (pMFC neutral condition: *M* = 4.39, *SD* = 1.92; MT/V5 neutral condition: *M* = 4.54, *SD* = 2.44), *P* = 0.815 (see Figure 1). However, there was no statistically significant interaction between the TMS and threat conditions, *P* = 0.207.

Planned contrasts also revealed that, consistent with the prediction that positive religious beliefs will increase in response to ‘alarming’ reminders of death absent pMFC downregulation, participants whose control MT/V5 region was downregulated produced higher ratings of positive religious beliefs in the death writing condition (*M* = 5.59, *SD* = 2.13) than in the neutral writing condition (*M* = 4.54, *SD* = 2.44), although this difference was not statistically significant using a two-tailed test, *P* = 0.099, 95% CI −2.30 to 0.20.

#### Negative religious beliefs.

Departing somewhat from the results of Holbrook *et al.*, (2016), in which a similar trend was observed but downregulatory TMS of pMFC did not significantly reduce avowed belief in negative religious concepts, planned contrasts revealed a significant effect of TMS within the subsample of participants who wrote about death (pMFC death condition: *M* = 3.68, *SD* = 1.71; MT/V5 death condition: *M* = 5.05, *SD* = 2.42), *F*(1, 49) = 5.41, *P* = 0.024, *η_p_^2^ *= 0.05, 95% CI 0.19–2.56 (see Figure 1). No effect of TMS on negative religious beliefs was observed in the neutral writing condition (pMFC neutral condition: *M* = 4.06, *SD* = 1.89; MT/V5 neutral condition: *M* = 4.40, *SD* = 2.44), *P* = 0.593 (see Figure 1). As with positive religious beliefs, the interaction between the TMS and threat conditions on affirmation of negative religious beliefs was not statistically significant, *P* = 0.241, despite the difference in the effects of TMS observed in the death writing *vs* neutral writing subsamples.

##### Group bias

Against predictions, a between-subjects ANOVA revealed no significant effects of the TMS manipulation, *P* = 0.558. Likewise, there were no effects of the threat manipulation, nor interactions between the two, on ratings of the critical author, *p*s 0.47–0.81, or the complimentary author, *p*s 0.17–0.75. This does not appear to owe to a failure of manipulation in regard to critique of the in-group, as a repeated-measures ANOVA (pooling conditions) confirmed that the critical student author was rated as significantly less appealing (*M* = 3.09, *SD* = 1.36) than the complimentary author (*M* = 5.03, *SD* = 1.36), *F*(1, 95) = 87.01, *P* < 0.001, *η_p_^2^ *= 0.48 (for descriptives, see Supplementary Table S3).

We explored whether differences in identification with the university might account for the null effects on group bias (6.3% reported no feeling of identification; 76% reported moderate identification; 17.7% reported extreme identification). Spearman’s ρs revealed no significant relationship between level of identification and assessments of the critical author (*r_s_* = −0.10, *P* = 0.352), and a positive correlation with assessments of the complimentary author (*r_s_* = 0.35, *P* = 0.001).

## Discussion

This pre-registered study replicated the prior finding that downregulating pMFC via cTBS reduces self-reported religious belief in the context of a recent, vivid reminder of death and bodily decomposition. Importantly, the addition of a non-threat control condition to the present design renders the reduction in religious belief more interpretable as an alarm effect. The results argue against an intrinsic relationship between pMFC and religious belief, or representation of religious concepts, as there were no such effects of the TMS manipulation in the non-threat writing condition. Notably, whereas downregulating pMFC did not significantly reduce avowed belief in negative religious ideas in the prior study, here we observed a parallel reduction in negative as well as positive beliefs in the death writing condition, presumably owing to the ideological links between reassuring afterlife concepts and corollary concepts such as Hell.

We have conceptualized the differential effects of downregulatory TMS observed in the death *vs* neutral threat conditions as reflecting an alarm-specific role of pMFC in religious belief. Nonetheless, although the death manipulation in the present study did somewhat heighten positive religious beliefs in the control TMS condition as predicted (*P* = 0.048, one-tailed), there were no significant interactions between the threat and TMS conditions, plausibly due to a lack of power. In light of the non-significant interactions, and the relatively modest effect of the threat manipulation on religiosity in the control TMS condition, the present results should be treated with caution. Nonetheless, the overall pattern of findings appears best construed as reflecting an alarm function of pMFC, given that religiosity in the death writing condition substantively decreased when pMFC was downregulated (replicating Holbrook *et al.*, 2016), and in consideration of the substantial evidence that reminders of death trigger heightened religiosity (e.g. Osarchuk and Tatz, 1973; Norenzayan and Hansen, 2006; Willer, 2009; Tracey *et al.*, 2011; Jong *et al.*, 2012; Vail *et al.*, 2012). Future studies utilizing larger samples should further clarify the extent to which downregulation of pMFC exerts different effects under threatening *vs* neutral contexts.

The generalizability of the present results to individuals invested with strong religious beliefs also requires further study. Our findings do not adjudicate whether an alarm role of pMFC would modulate levels of belief among a sample harboring strong prior religious or irreligious convictions, because we eliminated devoutly religious or atheistic individuals during the recruitment process. Relatedly, prior research associates religiosity with decreased sensitivity of medial frontal regions to performance errors (Inzlicht *et al.*, 2009; Inzlicht and Tullett, 2010), a subtype of the broad class of stimuli hypothesized to elicit compensatory responses, suggesting that downregulating pMFC might not alter religious convictions among devout participants for whom the prospect of death may arouse a muted alarm response. The ‘motivated meaning-making’ model of the function of religious conviction which inspired this prior research contends that devout individuals derive less anxiety from, and take less note of, anomalies, discrepancies or errors of various types that are mostly thematically unrelated to religious beliefs (Inzlicht, Tullet and Good, 2011). The present results harmonize with the meaning-making account insofar as pMFC appears to neurally mediate an alarm function that can invoke religious beliefs in response to perceived threats. However, the relationships between religiosity, alarm reactions and pMFC that we postulate are narrower in scope. We hypothesize that cues of a particular problem (e.g. death) registered by pMFC can engender a germane, palliative ideological response (e.g. afterlife beliefs), but make no broader claims regarding the putative function(s) of religiosity. While mechanisms correlated with religiosity may indeed attenuate alarm, at least in some contexts, the construct of ‘religion’ appears to reference an amalgamation of dissociable mechanisms that serve distinct functions and can coalesce into varying, culturally contingent assemblages (Boyer, 2003; McKay and Whitehouse, 2015).

Imaging research is necessary to clarify the relationship between pMFC activation and recruitment of neural mechanisms that correlate with uptakes in ideological adherence. In addition to specifying which regions (e.g. related to religious ideas) track activation of pMFC (e.g. upon being reminded of one’s death), imaging might also reveal which subcomponents of pMFC drive ideological effects, and connectomic analyses might illuminate how pMFC mechanisms articulate with other regions throughout the brain (Human Connectome Project, 2020). Causal interpretations of the functional role of pMFC must be approached cautiously pending this follow-up work, as TMS can generate spreading activation to regions that are proximal to or functionally downstream from the targeted region.

Departing from the findings of the prior study, we observed no effect of downregulating pMFC on group bias. Unfortunately, this result is difficult to interpret given the methodological shift from critique of the national in-group to critique of a university community. Participants in the present sample may not have cherished their collegiate identities comparably to the national identification used in the previous study, a speculation which appears consistent with the finding that identification with UCM did not significantly correlate with negative assessments of the critical author. Nonetheless, given that the critical student author character in the current study was evaluated more negatively than the complimentary student author character, yet no effect of TMS was observed, we can conclude that a putative effect of downregulating pMFC on group bias does not robustly generalize. However, because TMS effects occur due to an interaction between the stimulation and ongoing brain activity, the present null result of our TMS manipulation does not rule out the possibility that some neural activity in pMFC did evoke derogation of the critical author, but at a level too low to interact with TMS. In contrast, the critique of the national group in the prior study may have evoked stronger activity, rendering an effect of TMS detectable. Replication utilizing nationalistic group criticism stimuli is required to ascertain whether the observation of the effect of downregulating pMFC reported in Holbrook *et al.*, (2016) was a fluke.

## Conclusion

The pMFC appears to spur investment in relatively abstract beliefs to address relevant threats. This dynamic accords with the established role of pMFC in recruiting task-relevant responses to a broad array of perceptual and motor tasks (Navarrete *et al.*, 2004; Rushworth *et al.*, 2007; Danielmeier *et al.*, 2011), suggesting that functions evolved for adaptive responses to relatively concrete challenges have been extended in humans to invoke ideological shifts. Whether co-optation of the brain’s alarm system for ideological shifts reflects functional evolutionary adaptation in *Homo sapiens* (e.g. to foster cooperation related to shared ideology in the face of threat), or a by-product of the deployment of alarm systems in a mind capable of abstract ideological cognition, remains an open question.

## Supplementary Material

nsaa153_Supp

## Footnotes

1 The writing tasks and author evaluation methods were borrowed, with modification, from a body of work associated with Terror Management Theory (TMT), which focuses on putative effects of awareness of death on myriad facets of social judgment (e.g. Greenberg *et al.*, 1990). The theoretical tenets and empirical claims of TMT are highly contested (e.g. see Navarrete *et al.*, 2004; Jonas *et al.*, 2014; Holbrook, 2016). The present research is not motivated by TMT. Whereas TMT contends that supernatural beliefs culturally evolved to palliate death anxiety (e.g. Vail *et al.*, 2019), we favor approaches highlighting the multifaceted origins of supernatural beliefs (see McKay and Whitehouse, 2015) and only expect supernatural beliefs to be invoked to address the problem of death among individuals embedded in cultural contexts containing such palliative concepts as ‘Heaven’. Further, whereas TMS is premised on the notion that mortality cues uniquely influence social judgment, numerous studies have demonstrated comparable effects of death and non-death threat cues on social judgment (Jonas *et al.*, 2014).

2 In addition to the replication and extension efforts reported here with regard to religious belief and group bias, the study also included measures of empathy and mentalizing which are being prepared for separate publication, as they involve distinct theoretical considerations.

3 A computer error was discovered during data collection; 16 participants had inadvertently skipped one or more blocks. We corrected the issue and ran 16 further participants, producing a final sample just short of our pre-registered target of 100. However, the window for recruitment had closed and the total of 96 was deemed sufficient.
