# Supplementary material for: Posterior medial frontal cortex and threat-enhanced religious belief: a replication and extension
Source: Soc Cogn Affect Neurosci. 2020 Nov 12;15(12):1350–6. doi: 10.1093/scan/nsaa153 (PMC7759203; doi:10.1093/scan/nsaa153)
Supplement: nsaa153_Supp [file nsaa153_supp.zip › Supplementary.docx]

**Supplementary Online Material**

**to accompany**

**Posterior Medial Frontal Cortex and Threat-Enhanced Religious Belief:**

**A Replication and Extension**

Colin Holbrook, Marco Iacoboni, Chelsea Gordon, Shannon Proksch,

and Ramesh Balasubramaniam

**Table of Contents**

**Writing Tasks**

**Group bias Stimuli**

**Self-reported Affect**

**Complete cTBS Procedure**

**Neurological and Other TMS Contraindication Pre-screen**

**Figures:**

- **Figure S1.** Electrode placement areas used to target right pMFC (F2) or right MT/V5 (PO8).

**Tables:**

- **Table S1.** Self-reported affect by condition
- **Table S2.** Null effects of TMS or Threat manipulations on self-reported affect
- **Table S3.** Descriptive statistics for ratings of the personal qualities and arguments of the critical and complimentary authors

The full survey instrument and data are archived at <https://osf.io/ycjt8/>

**Writing Tasks**

Participants were randomly assigned to write brief responses on the neutral topic of their morning routine or on the subject of their own death.

*Neutral writing prompts:*

(a) “Please briefly describe the emotions that the thought of your morning routine arouses in you.”

(b) “Please jot down, as specifically as you can, what you think will happen to your body as you physically do your morning routine tomorrow.”

*Death writing prompts:*

(a) “Please briefly describe the emotions that the thought of your own death arouses in you.”

(b) “Please jot down, as specifically as you can, what you think will happen to your body as you physically die and once you are physically dead.”

This particular threat-induction was selected because of the evident link between the prospect of death and palliative thoughts of God and the afterlife, and also because this induction has been shown to heighten religiosity in prior studies (Jong et al., 2013; McGregor et al., 2013).

**Group Bias Stimuli**

*Complimentary student:*

The first thing that hit me when I came to UC Merced, was the incredible energy people had. Energy to go to school, energy to work to help put themselves through school, energy to be part of campus life. At this school people can train for the career they want. The classes are overall good quality with capable teachers, and can help students learn what they need to get ahead. Here anyone who work hard and studies hard can make their own success. At my old school, students do not have much motivation and hardly amount to much. At UC Merced, students have more opportunity than at almost any other school and success can be earned by anyone. While there are problems anywhere, UC Merced truly is a great school and I don’t regret my decisions to come here at all.

*Critical student:*

When I first came to UC Merced I believed it would be a great school but I soon realized it only seems great to people who don’t know any better. UC Merced is definitely not giving as good an education as the other UC schools. Many students do not seem ready to be successful after college. All people care about here is checking social media in class and hanging out with their friends. This not curiosity for learning. It’s all immature people and mediocre teachers who think they are doing a great job, but they’re not. A lot of UC Merced students just don’t seem that motivated. Also the campus is very divided into different groups that don’t really talk to each other and don't care about the campus community. It thinks it’s a great university but its not.

**Self-reported Affect**

Following the death writing task, participants completed the Positive and Negative Affect Schedule—Expanded Form (PANAS-X; Watson and Clark, 1991). The PANAS-X measures consciously accessible affect by asking participants to rate the extent to which they feel 60 affective states, with subscales assessing general Positive (α = .93) and Negative (α = . 88) affect, as well as specific negative emotions (Fear [α = . 84], Hostility [α = . 83], Guilt [α = . 89], Sadness [α = . 89]), positive emotions (Joviality [α = . 95], Self-assurance [α = . 90], Attentiveness [α = . 76]), and other affective states (Shyness [α = . 72], Fatigue [α = . 85], Serenity [α = . 81], and Surprise [α = . 76]). Participants rated their current affective states using a 5-point Likert scale (1 = *Not at all*; 2 = *A little;* 3 = *Moderately*; 4 = *Quite a bit*; 5 = *Extremely*; for descriptives, see Table S1).

**Complete cTBS Procedure**

Seat participant in a comfortable position and fit a grid-marked swim cap to his/her head. Follow the steps below based on relevant control vs treatment TMS conditions.

Control Condition (MT/V5 – Right Occipital Cortex)

- Attach two differential parallel-bar EMG recording electrodes, centered over the belly of the right first dorsal interosseous muscle on the hand, after cleaning the skin over the muscle thoroughly. Attach a third, ground electrode to the skin over a bone on the elbow. Have the subject rest comfortably and begin recording the EMG electrode output to software that will filter and display the signal.
- Mark the target location (MT/V5, electrode placement PO8, for the control condition) on the swim cap using the 10-20 system following these steps:
  - Measure nasion to inion, make note of the total (in cm), and make marks at 10% of this measure (Fp), 50% (Cz), 90% (O).
  - Measure right to left tragus, through Cz, making note of the total (in cm) and make marks at 10% (T4).
  - Measure the right partial circumference from Fp to O, through T4, and make a mark at

80% (1/2 way between T6 and O2). This is the target location for MT/V5.

- Perform thresholding on the left primary motor cortex. With a figure-eight coil (70 mm), place the center of the coil over motor cortex, held tangential to the scalp surface and oriented at an angle of ~45 degrees from the anterior-posterior midline. Apply single-pulse TMS at 50% of maximum stimulus output (MSO) and observe whether a motor-evoked potential (MEP) was present in the EMG signal following stimulation.
- If no MEP is seen following stimulation, reposition the coil one centimeter away in any direction and try stimulation again. Wait at least 6-10 seconds between stimulations in order for neurons to completely recover. Continue moving the coil one centimeter at a time, marking on the cap the stimulation sites that result in an MEP of 50 µV or greater. If no MEPs are seen after trying many locations, increase the stimulation intensity by 5% at a time, until MEPs are observed.
- Multiple nearby locations on the grid may elicit reliable MEPs. If this is the case, stimulate each of these locations at intensities decreasing by 1% at a time, until only one location remains that elicits reliable MEPs.
- To determine active motor threshold (aMT), the subject is asked to flex the FDI muscle by squeezing a stress ball. While the muscle is contracted, stimulate the located region for 10 repetitions, separated by ~7 seconds, at decreasing intensities, until a corresponding observable twitch in the hand muscle no longer occurs for 50% of stimulations (5 out of 10). The threshold is the lowest intensity that elicits 5/10 visible twitches, verified by visually comparing MEP size and consistency. High concordance has been shown between using threshold estimations determined with electromyography and visual twitch (Stokes et al, 2005; Pridmore, Ferandes Filho, Nahas, Liberatos & George, 1998) and visual twitch is often used to determine AMT (Sandrini, Umiltà, & Rusconi, 2011; Göbel, Calabria, Farnè, & Rossetti, 2006; Göbel, Walsh, & Rushworth, 2001).
- Navigate the coil to the target location on the cap corresponding to the location marked as MT/V5. Face the coil directly to the front of the head and oriented parallel with the anterior-posterior midline.
- Apply cTBS as follows: three pulses at 50 Hz repeated at 200 milliseconds (ms) intervals for 40 seconds, totaling 600 pulses.

Treatment Condition (pMFC – Right Frontal Cortex)

- Attach two differential parallel-bar EMG recording electrodes, centered over the belly of the right tibialis anterior muscle on the leg, after cleaning the skin over the muscle thoroughly. Attach a third, ground electrode to the skin over a bone on the ankle. Have the subject rest comfortably and begin recording the EMG electrode output to software that will filter and display the signal.
- Mark the target location (mPFC, electrode placement F2, for the treatment condition) on the swim cap using the 10-20 system following these steps:
  - Measure nasion to inion, make note of the total (in cm), and make marks at 10% of this measure (Fp), 30% (Fz), 50% (Cz), and 90% (O).
  - Measure right to left tragus, through Cz, making note of the total (in cm) and make marks at 10% (T4).
  - Measure the right partial circumference from Fp to O, through T4, and make a mark at 30% (F8).
  - Measure from F8 to Fz and make a mark at 75% of this measure. This is the target location for pMFC (which is in effect 1/2 between Fz and F4, and should be ~3.75 cm anterior to motor cortex).
- Perform thresholding on the left primary motor cortex. With double-cone coil (110mm) place the center of the coil over the midline. Apply single-pulse TMS at 50% of maximum stimulus output (MSO) and observe whether a motor-evoked potential (MEP) was present in the EMG signal following stimulation.
- If no MEP is seen following stimulation, reposition the coil one centimeter away in any direction and try stimulation again. Wait at least 6-10 seconds between stimulations in order for neurons to completely recover. Continue moving the coil one centimeter at a time, marking on the cap the stimulation sites that result in an MEP of 50 µV or greater. If no MEPs are seen after trying many locations, increase the stimulation intensity by 5% at a time, until MEPs are observed.
- Multiple nearby locations on the midline may elicit reliable MEPs. If this is the case, stimulate each of these locations at intensities decreasing by 1% at a time, until only one location remains that elicits reliable MEPs.
- To determine active motor threshold (aMT), the subject is asked to contract the TA muscle by placing their heel firmly on the ground and pointing their toes upward. While the muscle is contracted, stimulate the located region for 10 repetitions, separated by ~7 seconds, at decreasing intensities, until a corresponding MEP of at least 100 µV peak to peak greater than the preceding EMG signal during muscle contraction is observed 50% of the time (5 out of 10), verified by visually comparing MEP size and consistency.
- Due to a number pilot participants reporting significant pain during cTBS over pMFC using the double-cone coil, any participant with an AMT above 40% of maximal stimulus output is excluded and does not undergo cTBS and is excluded from the experiment.
- Navigate the coil to the target location on the cap corresponding to the location marked as pMFC.
- Apply cTBS as follows: three pulses at 50 Hz repeated at 200 milliseconds (ms) intervals for 40 seconds, totaling 600 pulses.

**
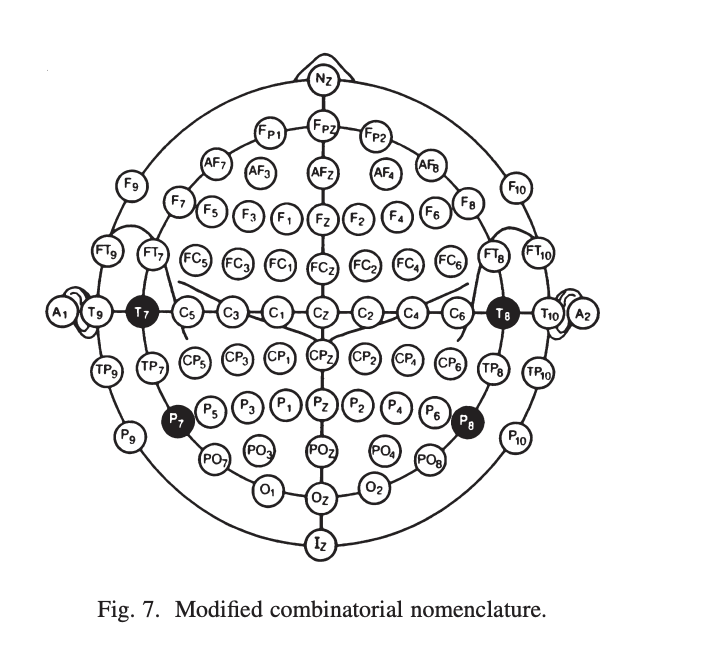
**

Right MT/V5 target site

Right pMFC target site

**Figure S1.** Electrode placement areas used to target right pMFC (F2) or right MT/V5 (PO8). (Image modified with permission from Klem, Luders, Jasper, & Elger, 1999.)

**Neurological and Other TMS Contraindication Pre-screen**

1. YES/NO – Are you younger than 18 years old?
2. YES/NO – Are you dominantly left-handed or ambidextrous?
3. YES/NO – Do you have a pacemaker or intracardiac lines?
4. YES/NO – Do you have any metal/electrical/magnetic objects implanted in your body except dental fillings?
5. YES/NO – Do you have an implanted neurostimulator (e.g. DBS, epidural/subdural, VNS)?
6. YES/NO – Are you or could you possibly be pregnant?
7. YES/NO – Do you have any major medical problems or unstable medical problems?
8. YES/NO – Do you have any history of neurological or psychiatric illness?
9. YES/NO – Have you had any head injury or head surgery?
10. YES/NO – Do you have any poorly controlled migraine headaches?
11. YES/NO – Do you or any blood relatives (grandparent, parent, aunt/uncle, sibling, self) have a history of a seizure disorder? If so, please circle all relations that apply.
12. YES/NO – Have you ever had a convulsion or a seizure?
13. YES/NO – Have you had unusual responses, such as fainting, when you go to the hospital or get blood drawn?
14. YES/NO – Have you ever had a fainting spell or syncope (passing out)?
15. YES/NO – Have you ever had a surgical procedure to your spinal cord?
16. YES/NO – Do you have spinal or ventricular derivations?
17. YES/NO – Do you regularly take any medications or use street drugs? If so, please discuss the exact medications you are taking with the researcher.
18. YES/NO – Have you taken any medication other than contraceptives in the past 12 hours?
19. YES/NO – Have you used alcohol, nicotine, or drugs in the past 12 hours?
20. YES/NO – Do you have a medication infusion device?
21. YES/NO – Do you have any metal on your body (watch or jewelry, hair holders or pins, eyeglasses, body piercings, wallet, keys)? If so, please remove.
22. YES/NO – Do you have atypical hearing?
23. YES/NO – Do you have any hearing problems or ringing in your ears?

To ensure participant safety, participants must be able to answer "No" to all questions. The participant fills out a pre-screening form online before being signed up to participate in a TMS study. The pre-screening form is filled out again on the day of the TMS study during the informed consent process, and after the researcher has explained the safety and risks of participating in a TMS procedure.

**Supplementary Table S1**

**Self-reported Affect by Condition**

|  | pMFC  Neutral | | pMFC  Death | | | | MT/V5  Neutral | | MT/V5  Death | | |  |  |
| --- | --- | --- | --- | --- | --- | --- | --- | --- | --- | --- | --- | --- | --- |
|  | (*N* = 24) | | (*N* = 25) | | | | (*N* = 21) | | (*N* = 26) | | |  |  |
|  | *M* | *SD* | | *M* | *SD* | | *M* | *SD* | | *M* | *SD* | |  |
| Positive Affect | 2.48 | .95 | | 2.12 | | .69 | 2.57 | .95 | | 2.30 | .93 | | |
| Negative Affect | 1.35 | .57 | | 1.56 | | .63 | 1.47 | .71 | | 1.54 | .57 | | |
| Fear | 1.40 | .55 | | 1.72 | | .67 | 1.56 | .90 | | 1.68 | .60 | | |
| Hostility | 1.27 | .58 | | 1.39 | | .60 | 1.35 | .64 | | 1.30 | .39 | | |
| Guilt | 1.29 | .67 | | 1.46 | | .74 | 1.37 | .66 | | 1.44 | .67 | | |
| Sadness | 1.39 | .72 | | 1.75 | | .81 | 1.54 | .83 | | 1.64 | .75 | | |
| Joviality | 2.34 | 1.04 | | 1.93 | | .69 | 2.43 | 1.00 | | 2.14 | 1.00 | | |
| Self-Assurance | 2.33 | .95 | | 1.87 | | .64 | 2.32 | 1.02 | | 2.12 | .94 | | |
| Attentiveness | 2.72 | .87 | | 2.58 | | .79 | 3.01 | 1.02 | | 2.60 | .88 | | |
| Shyness | 1.41 | .42 | | 1.59 | | .70 | 1.74 | .83 | | 1.59 | .53 | | |
| Fatigue | 2.41 | 1.03 | | 3.11 | | .80 | 2.60 | 1.01 | | 2.64 | 1.06 | | |
| Serenity | 3.44 | .84 | | 3.08 | | .94 | 3.50 | .85 | | 3.36 | .97 | | |
| Surprise | 2.03 | .98 | | 1.69 | | .69 | 2.03 | .93 | | 1.67 | .81 | | |

*Note.* *N* = 96. Subjective affect was measured using the Positive and Negative Affect Schedule—Expanded Form (Watson & Clark, 1991).

**Supplementary Table S2**

**Null Effects of TMS or Threat Manipulations on Self-reported Affect**

|  | Threat | | | TMS | | | |  |
| --- | --- | --- | --- | --- | --- | --- | --- | --- |
|  | *F* | *p* | *η_p_^2^* | | *F* | *p* | *η_p_^2^* | |
| Positive Affect | .57 | .452 | .01 | | 2.97 | .091 | .03 | |
| Negative Affect | .17 | .681 | .00 | | 1.41 | .239 | .02 | |
| Fear | .15 | .695 | .00 | | 2.47 | .120 | .03 | |
| Hostility | .00 | .976 | .00 | | .08 | .775 | .00 | |
| Guilt | .05 | .828 | .00 | | .82 | .369 | .01 | |
| Sadness | .01 | .912 | .00 | | 2.08 | .152 | .02 | |
| Joviality | .55 | .460 | .01 | | 3.25 | .075 | .03 | |
| Self-Assurance | .43 | .514 | .01 | | 3.14 | .079 | .03 | |
| Attentiveness | .72 | .397 | .01 | | 2.32 | .131 | .03 | |
| Shyness | 1.69 | .197 | .02 | | .02 | .881 | .00 | |
| Fatigue | .51 | .477 | .01 | | 3.43 | .067 | .04 | |
| Serenity | .81 | .369 | .01 | | 1.86 | .176 | .02 | |
| Surprise | .00 | .963 | .00 | | 3.94 | .050 | .04 | |

*Note.* *N* = 96. Subjective affect was measured using the Positive and Negative Affect Schedule—Expanded Form (Watson & Clark, 1991). There were no significant main effects, nor interactions between the TMS or Threat manipulations, on any of the state affect subscales, *p*s .10 - .95.

**Supplementary Table S3**

**Descriptive Statistics for Ratings of the Personal Qualities and Arguments of the Critical and Complimentary Authors**

|  | pMFC  Neutral | | pMFC  Death | | | | MT/V5  Neutral | | | MT/V5  Death | | |  |
| --- | --- | --- | --- | --- | --- | --- | --- | --- | --- | --- | --- | --- | --- |
|  | (*N* = 24) | | (*N* = 25) | | | | (*N* = 21) | | | (*N* = 26) | | |  |
|  | *M* | *SD* | *M* | *SD* | | *M* | | *SD* | *M* | | *SD* |  |  |
| *Critical* |  |  |  | |  |  | |  |  | |  | | |
| Personal | 3.33 | 1.27 | 3.11 | | 1.13 | 2.89 | | 1.31 | 3.25 | | 1.66 | | |
| Arguments | 3.02 | 1.99 | 3.06 | | 1.96 | 2.81 | | 1.68 | 2.92 | | 1.70 | | |
| Total | 3.23 | 1.41 | 3.09 | | 1.27 | 2.87 | | 1.24 | 3.13 | | 1.51 | | |
| *Complimentary* |  |  |  | |  |  | |  |  | |  | | |
| Personal | 5.06 | 1.07 | 4.74 | | 1.35 | 5.10 | | 1.56 | 5.37 | | 1.45 | | |
| Arguments | 5.23 | 1.15 | 4.46 | | 1.83 | 4.83 | | 2.22 | 5.23 | | 1.52 | | |
| Total | 5.12 | .98 | 4.65 | | 1.41 | 5.03 | | 1.62 | 5.32 | | 1.37 | | |

*Note.* *N* = 96. There were no significant main effects of the TMS or Threat manipulations, interactions between the two, nor contrasts between cells, *p*s .08 - .99.

References

Göbel, S.M., Calabria, M., Farnè, A., Rossetti, Y., (2006). Parietal rTMS distorts the mental

number line: simulating ‘spatial’ neglect in healthy subjects. *Neuropsychologia, 44*, 860–868.

Göbel, S., Walsh, V., Rushworth, M.F., 2001. The mental number line and the human angular

gyrus. *Neuroimage, 14*, 1278–1289.

Jong, J., Halberstadt, J., & Bluemke, M. (2013). Foxhole atheism, revisited: The effects of

mortality salience on explicit and implicit religious belief. *Journal of Experimental Social Psychology*, *48,* 983–989.

Klem, G.H., Luders, H.O., Jasper, H.H., Elger, C. (1999). The ten-twenty electrode system of the International Federation. *Electroencephalography and Clinical Neurophysiology Supplement, 52,* 3–6.

McGregor, I., Prentice, M., & Nash, K. (2013). Anxious uncertainty and reactive approach

motivation (RAM) for religious, idealistic, and lifestyle extremes. *Journal of Social Issues*, *69,* 537-563.

Pridmore, S., Fernande Filho, J. A., Nahas, Z., Liberatos, C., & George, M.S., (1998). Motor

threshold in transcranial magnetic stimulation: a comparison of a neurophysiological method and a visualization of movement method. *The Journal of ECT,* *14*, 25–27.

Sandrini, M., Umiltà, C., Rusconi, E., (2011). The use of transcranial magnetic stimulation in

cognitive neuroscience: A new synthesis of methodological issues. *Neuroscience and Biobehavioral Reviews, 35,* 516-536.

Stokes, M. G., Chambers, C. D., Gould, I. C., Henderson, T. R., Janko, N. E., Allen, N. B.,

& Mattingley, J. B., (2005). Simple metric for scaling motor threshold based on scalp-cortex distance: application to studies using transcranial magnetic stimulation. *The Journal of Neurophysiology, 94,* 4520-4527.

Watson, D., & Clark, L.A. (1991). *Preliminary manual for the PANAS-X: Positive and Negative*

*Affect Schedule—Expanded Form.* Unpublished manuscript, Southern Methodist University, University Park, TX.
